# Особенности применения синтетических аналогов гормонов щитовидной железы: результаты международного исследования 2020 THESIS* среди членов Белорусского общественного медицинского объединения «Эндокринология и метаболизм»

**DOI:** 10.14341/probl12828

**Published:** 2021-12-06

**Authors:** А. П. Шепелькевич, Ю. В. Дыдышко, Е. B. Юреня, В. Л. Лобашова, Р. Аттанасио, Л. Хегедюс, Э. Надь, Р. Негро, Э. Папини, П. Перрос

**Affiliations:** Белорусский государственный медицинский университет; Белорусский государственный медицинский университет; Республиканский центр медицинской реабилитации и бальнеолечения; Минский городской клинический эндокринологический центр; Республиканский центр медицинской реабилитации и бальнеолечения; Ортопедический институт Галеацци,; Университетская больница Оденсе; Университет Дебрецен; Больница им. В. Фацци; Больница Регины Апостолорум; Больница Королевы Виктории

**Keywords:** левотироксин, лиотиронин, гипотиреоз, эутиреоз, анкетирование

## Abstract

**ОБОСНОВАНИЕ:**

ОБОСНОВАНИЕ. Стандартным лечением гипотиреоза является заместительная терапия левотироксином (LT4), который в Республике Беларусь доступен в форме таблеток, тогда как лиотиронин (LT3) не зарегистрирован, но пациенты могут приобретать его самостоятельно.

**ЦЕЛЬ:**

ЦЕЛЬ. Данное исследование было направлено на изучение опыта применения белорусскими эндокринологами синтетических аналогов гормонов щитовидной железы у пациентов с гипотиреозом и эутиреозом.

**МАТЕРИАЛЫ И МЕТОДЫ:**

МАТЕРИАЛЫ И МЕТОДЫ.  Проводилось онлайн-анкетирование, для участия в котором были приглашены члены Белорусского медицинского общественного объединения «Эндокринология и метаболизм» (БОМО «Эндокринология и метаболизм») посредством размещения информации в групповом чате мессенджера Viber и по электронной почте. Период проведения исследования – с 1-го октября по 26-е декабря 2020 года. Было получено 210 анкет, 146 из которых были использованы для дальнейшего анализа.

**РЕЗУЛЬТАТЫ:**

РЕЗУЛЬТАТЫ. Подавляющее количество участников исследования 145 (99,3%) человек указали, что используют LT4 в качестве препарата первого выбора для терапии пациентов с гипотиреозом. 61 (41,8%) врачей ответили, что, вероятно, комбинированная терапия LT3+LT4 может быть использована у пациентов с длительно нелеченым гипотиреозом и 15 (10,3%) ‒ у пациентов со стойкими симптомами гипотиреоза, несмотря на биохимический эутиреоз на терапии LT4. Более половины респондентов 84 (57,5%) указали, что терапия LT4 не показана пациентам с эутиреозом, однако 50 (34,2%) врачей рассматривали возможность ее назначения при женском бесплодии с высоким уровнем тиреоидных антител и 36 (24,7%)  –  при простом нетоксическом зобе, имеющем тенденцию к увеличению. В различных клинических ситуациях большинство белорусских эндокринологов для лечения гипотиреоза предпочитали таблетки иным лекарственным формам LT4 и не ожидали каких-либо серьезных различий при переходе на препарат другого типа. Однако меньшая часть респондентов указала на возможность использования новых составов LT4 в ситуациях с предположительно сниженной абсорбцией или биодоступностью таблетированной формы LT4 (таблетки + «не ожидаю серьезных изменений с разными формами» vs. «мягкие гелевые капсулы» + «жидкий раствор»; p <0,001). Стойкие симптомы гипотиреоза на фоне заместительной терапии LT4 с достижением целевого ТТГ преимущественно могут быть вызваны психосоциальными факторами, сопутствующими заболеваниями, нереалистичными ожиданиями пациента, синдромом хронической усталости, бременем хронического заболевания.

**ЗАКЛЮЧЕНИЕ:**

ЗАКЛЮЧЕНИЕ. Методом выбора белорусских эндокринологов в лечении гипотиреоза является заместительная терапия LT4, но назначение комбинированной терапии LT4+LT3 может быть рассмотрено в отдельных клинических ситуациях. Как правило, эндокринологи не назначают LT4 пациентам с эутиреозом и не ожидают существенной разницы при применении других форм левотироксина.

## Abstract (en)

**BACKGROUND:**

BACKGROUND. The standard treatment for hypothyroidism is levothyroxine (LT4), which in the Republic of Belarus is available in tablet form whereas liothyronine (LT3) is not registered, but patients can purchase them on their own abroad.

**AIM:**

AIM. This study aimed to investigate Belarusian endocrinologists’ attitude of thyroid hormones in hypothyroid and euthyroid patients.

**MATERIALS AND METHODS:**

MATERIALS AND METHODS. An online survey was conducted, for which members of the Belarusian Medical Association of Endocrinology and Metabolism were invited by posting information in the group chat and by e-mail. The research period was from October 1 to December 26, 2020. 210 questionnaires were received, 146 of which were used.

**RESULTS:**

RESULTS. The majority of participants, 145 (99.3%), indicated that they were using LT4 as the first-choice drug for the treatment hypothyroidism. Sixty-one (41.8%) doctors answered that LT3 + LT4 combination likely can be used in patients with long-term untreated hypothyroidism and 15 (10.3%) — in patients with persistent symptoms of hypothyroidism, despite biochemical euthyroidism on therapy LT4. Over half of the respondents 84 (57.5%) answered that thyroid hormone therapy was never indicated for euthyroid patients, but 50 (34.2%) would consider it for female infertility with high level of thyroid antibodies and 36 (24.7%) for simple goiter growing over time. In various conditions that could interfere with absorption of LT4, most responding Belarusian endocrinologists do not expect a significant difference with different formulations (tablets

+“I do not expect major changes in different forms” vs. “soft gel capsules”+“liquid solution”; p <0.001). Persistent symptoms of hypothyroidism on the background of LT4 replacement therapy with the achievement of the target TSH can mainly be caused by psychosocial factors, concomitant diseases, unrealistic patient expectations, chronic fatigue syndrome, and the burden of chronic disease.

**CONCLUSION:**

CONCLUSION. The method of choice of Belarusian endocrinologists in the treatment of hypothyroidism is LT4 replacement therapy, but the appointment of LT4 + LT3 combination therapy can be considered in certain clinical situations. As a rule, endocrinologists do not prescribe LT4 in patients with euthyroidism and do not expect a significant difference when using other forms of levothyroxine.

## ОБОСНОВАНИЕ

Гипотиреоз является распространенным заболеванием, которое выявляется практически у 3% европейского населения [1]. Назначение синтетических аналогов гормонов щитовидной железы, в частности левотироксина (LT4) в таблетированной форме, является стандартной терапией гипотиреоза. В последние годы разработаны и в ряде стран внедрены в клиническую практику другие формы выпуска препаратов LT4, такие как мягкие гелевые капсулы и растворы. Данные лекарственные формы были изготовлены с целью преодоления проблемных аспектов существующего лечения, связанного с возможным снижением его эффективности при одновременном приеме таблеток с едой и напитками, с некоторыми типами лекарств или при наличии сопутствующих желудочно-кишечных состояний, таких как инфекция Helicobacter pylori, целиакия, бариатрическая хирургия и атрофический гастрит [2].

Наличие высокой распространенности коморбидности у пациентов с гипотиреозом оказывает значимое влияние на своевременное выявление, а также эффективность и безопасность лечения. Установлено, что на момент постановки диагноза «гипотиреоз» распространенность сопутствующих соматических заболеваний [3] и психиатрических расстройств выше, чем у эутиреоидных лиц контрольной группы [4]. Медико-социальные последствия гипотиреоза ассоциированы с более высокой степенью утраты трудоспособности и выходом на инвалидность, снижением работоспособности и потерей в заработной плате [5], а также повышенной смертностью как от естественных, так и от внешних причин [6][7]. Кроме того, получены убедительные данные о снижении качества жизни пациентов, несмотря на проводимое лечение LT4 [8].

Учитывая вышеизложенное, использование других форм LT4, таких как раствор или мягкие гелевые капсулы, демонстрирующие различную биодоступность, особенно при нарушении абсорбции, может представлять собой клинически значимую альтернативу [2][9][10]. В то же время мягкие гелевые капсулы и раствор обладают более высокой стоимостью по сравнению с таблетированной формой, поэтому рентабельность их назначения остается нерешенной [11].

В международных и национальных клинических рекомендациях по лечению заболеваний щитовидной железы отдается предпочтение LT4 в качестве стандартной заместительной терапии, в то время как взгляды на комбинированную (LT4+LT3 (лиотиронин)) терапию расходятся [12][13]. В соответствии с рекомендациями Консенсуса 2021 г. по доказательному использованию комбинаций LT4+LT3 при лечении гипотиреоза основными аргументами в пользу назначения LT4+LT3 являются сохранение симптомов гипотиреоза и применение высоких доз LТ4 у ряда пациентов [13].

Кроме того, в ряде исследований активно обсуждаются психологические аспекты, связанные с недовольством пациентов, получающих монотерапию LT4, что также рассматривается в качестве дополнительных факторов для возможности назначения комбинированной терапии LT4+LT3 или даже препаратов из высушенной ткани щитовидной железы (ВЩЖ) [14][15]. В то же время назначение LТ3 сопряжено с потенциальными рисками, связанными с высокой вариабельностью уровней трийодтиронина и тиреотропного гормона (ТТГ) на фоне применения имеющихся комбинированных лекарственных средств, что требует создания пролонгированных форм LТ3 [12].

## ЦЕЛЬ ИССЛЕДОВАНИЯ

Целью исследования было изучение отношения белорусских эндокринологов к назначению синтетических аналогов гормонов щитовидной железы у пациентов с нормальной и сниженной функцией щитовидной железы.

## МАТЕРИАЛЫ И МЕТОДЫ

Место и время проведения исследования

Место проведения. Настоящее анкетирование проведено в Республике Беларусь и является частью продолжающегося международного исследования THESIS (Treatment of Hypothyroidism in Europe by Specialists: An International Survey, «Лечение гипотиреоза в Европе специалистами: международное анкетирование»).

Время исследования. Рассылка анкет проводилась трижды: первый раз 1 октября 2020 г. с последующими двумя напоминаниями. Анкетирование было завершено 26 декабря 2020 г.

Изучаемые популяции (одна или несколько)

Для участия в анкетировании были приглашены члены БОМО «Эндокринология и метаболизм» (www.endocrinology.by) посредством размещения информации в групповом чате мессенджера Viber и по электронной почте в форматах Word и Excel. Заполненные анкеты были получены координаторами проекта по электронной почте. Для обработки результатов все сведения занесены в итоговую таблицу в формате Excel.

Критерии включения: для дальнейшего анализа были включены анкеты с полностью заполненным разделом А, что соответствовало рекомендациям научной группы международного исследования THESIS.

Способ формирования выборки

Выборка участников исследования была сформирована путем сплошного включения наблюдений, соответствовавших критериям включения.

Дизайн исследования

Было проведено одномоментное исследование методом анкетирования.

Анкета, используемая в исследовании, была разработана научной группой международного исследования THESIS (Treatment of Hypothyroidism in Europe by Specialists: An International Survey) для оценки отношения европейских врачей-эндокринологов к лечению гипотиреоза и возможности применения синтетических аналогов гормонов щитовидной железы у эутиреоидных лиц.

Методом обратного перевода вопросы анкеты были переведены на русский язык и адаптированы для белорусских специалистов. Анкета включала два блока вопросов. Раздел А: 8 вопросов о демографических данных; раздел В: 23 вопроса о лечении пациентов с гипотиреозом и эутиреозом. В конце анкеты также было предусмотрено место для комментариев (вопрос 24) (приложение 1). Процесс анкетирования занимал 5–10 мин для одного врача.

Методы

Для дальнейшего анализа были включены анкеты с полностью заполненным разделом А, согласно рекомендациям научной группы международного исследования THESIS.

Статистический анализ

Для ответов на все вопросы использованы методы описательной статистики. Только респонденты, ответившие на все вопросы о демографических данных, считались пригодными для статистического анализа. Во всех анализах респонденты, заявившие, что они не знали ответа на данный вопрос, были объединены в категорию ответов с респондентами, которые не дали ответа. Для сравнения распределения частот между категориальными переменными был использован χ2-критерий. Также χ2 Пирсона использовался для проверки того, были ли переменные в демографических данных (раздел A) независимыми от результата в вопросах в разделе B. Если какая-либо переменная не была независимой от результата в любом вопросе в разделе B, выполнялся анализ логистической регрессии. Значение p<0,05 считалось статистически значимым. Все анализы проводились с использованием статистического программного обеспечения IBM SPSS версии 25 (SPSS Inc., Чикаго, Иллинойс, США).

Этическая экспертиза

Согласно заключению Комитета по этике Учреждения здравоохранения «Городской эндокринологический диспансер», исследование одобрено к проведению (г. Минск, Протокол №2 от 09.09.2020).

## РЕЗУЛЬТАТЫ

Характеристики респондентов

В течение почти 3 мес проведения анкетирования было получено 210 анкет, 146 из которых были использованы для дальнейшего анализа. Все респонденты являлись членами БОМО «Эндокринология и метаболизм», кроме того, трое участников (2,1%) также были членами Европейской тиреоидной ассоциации.

Демографические данные участников анкетирования представлены в таблице 1.

**Table:** Таблица 1. Параметры респондентов из Беларуси, принявших участие в международном исследовании 2020 THESIS*

Параметр	n (%)
Пол
Женский	132 (90,4)
Мужской	14 (9,6)
Возраст, лет
20–30	13 (8,9)
31–40	45 (30,8)
41–50	48 (32,9)
51–60	26 (17,8)
61–70	13 (8,9)
70+	1 (0,7)
Годы медицинской практики
0–10	37 (25,3)
11–20	35 (24,0)
21–30	46 (31,5)
31–40	22 (15,1)
40+	6 (4,1)
Специальностьa
Эндокринология	146 (100,0)
Детская эндокринология	16 (11,0)
Место работыa	
Университетская клиника	8 (5,5)
Государственная клиника	130 (89,0)
Частная клиника	8 (5,5)
Общеврачебная практика	7 (4,8)
Научный исследователь	4 (2,7)
Специализированная практика	6 (4,1)

Подавляющее большинство среди ответивших респондентов составляли женщины — 132 (90,4%) человека. Врачи-эндокринологи, принявшие участие в исследовании, лечили пациентов с патологией щитовидной железы еженедельно — 126 (86,3%) человек, 16 (11%) — ежедневно, и только 4 (2,7%) респондента редко работали с патологией щитовидной железы.

Как правило, среди участников анкетирования 116 (79,5%) респондентов оказывали медицинскую помощь более 100 пациентам с гипотиреозом ежегодно; 14 (9,6%) — 51–100 пациентам ежегодно, 11 (7,5%) — 10–50 пациентам в год, и только 5 (3,4%) врачей редко работали с гипотиреозом.

Лечение пациентов с гипотиреозом

Подавляющее количество участников исследования — 145 (99,3%) человек указали, что используют LT4 в качестве препарата первого выбора для терапии пациентов с гипотиреозом, однако 1 (0,7%) участник предпочитал комбинированную терапию LT4+LT3. Никто из врачей-эндокринологов не предлагал монотерапию ВЩЖ или LT3 в качестве первого выбора лечения (LT4 по сравнению с другими схемами терапии гипотиреоза; p<0,001).

Использование разных лекарственных форм LT4

При составлении анкеты экспертами научной группы THESIS был сформирован блок вопросов (В5–В9), посвященный оценке использования различных форм выпуска LT4 в конкретных клинических ситуациях (табл. 2).

**Table:** Таблица 2. Предпочтения респондентов из Беларуси, принявших участие в международном исследовании 2020 THESIS*, при выборе лекарственных форм левотироксина в различных клинических ситуациях

Факторы, влияющие на стабильность терапии	Я не ожидаю серьезных изменений с разными формами, n (%)	Таблетки/препарат другого производителя, n (%)	Мягкие гелевые капсулы,n (%)	Жидкий раствор,n (%)	Не уверен/нет ответа, n (%)
Какой препарат LT4, по вашему мнению, наименее подвержен переменному усвоению?	40 (27,4)	94 (64,4)	6 (4,1)	6 (4,1)	0 (0,0)
Какие из следующих форм препаратов LT4 вы бы прописали в случае впервые выявленного гипотиреоза, когда пациент сам сообщает о непереносимости различных продуктов, что повышает вероятность целиакии, мальабсорбции, непереносимости лактозы или непереносимости общих вспомогательных веществ?	31 (21,2)	81 (55,5)	21 (14,4)	21 (14,4)	0 (0,0)
Какие из следующих форм препаратов LT4 вы бы прописали пациенту, принимающему LT4, но у которого необъяснимо плохой лабораторный контроль гипотиреоза?	25 (17,1)	115 (78,8)	11 (7,5)	7 (4,8)	0 (0,0)
Какие из следующих препаратов LT4 вы бы прописали пациенту с плохим биохимическим контролем, который не может (из-за напряженного образа жизни) принимать LT4 натощак и отдельно от еды/питья?	49 (33,6)	66 (45,2)	14 (9,6)	17 (11,6)	0 (0,0)
Какой из следующих форм препаратов LT4 вы бы прописали пациенту, принимающему таблетки LT4, который имеет хороший лабораторный контроль гипотиреоза, но сохраняются клинические симптомы?	77 (52,7)	65 (44,5)	6 (4,1)	3 (2,1)	0 (0,0)

Результаты анкетирования показывают, что в различных клинических ситуациях большинство белорусских эндокринологов для лечения гипотиреоза предпочитали таблетки иным лекарственным формам LT4, таким как мягкие гелевые капсулы или жидкий раствор. Как правило, белорусские врачи не ожидали каких-либо серьезных различий при переходе с одного типа препарата на другой. Аналогичное отношение применимо к ситуациям с лекарственными взаимодействиями, непереносимостью различных продуктов, необъяснимо плохим контролем заболевания или стойкими его симптомами, несмотря на достижение целевых значений ТТГ.

В то же время меньшая часть ответивших членов БОМО «Эндокринология и метаболизм» указали на возможность использования новых составов LT4 в ситуациях с предположительно сниженной абсорбцией или биодоступностью таблетированной формы LT4 (таблетки + «не ожидаю серьезных изменений с разными формами» vs «мягкие гелевые капсулы» + «жидкий раствор»; p<0,001). Результаты логистического регрессионного анализа не выявили различий предпочтений лекарственной формы LT4 по возрасту респондентов или длительности врачебной практики.

Мониторинг эффективности заместительной терапии гипотиреоза

Согласно полученным ответам, после начала применения LT4 36 (24,7%) эндокринологов повторно назначали контроль уровня ТТГ в сыворотке через 4–6 нед, и 109 (74,7%) делали это через 8 нед. Только 1 (0,7%) участник указал, что возможен повторный контроль ТТГ уже через 2 нед, также никто из респондентов не предлагал оценивать только клинические данные для эффективности лечения.

При переходе на другую лекарственную форму или на препарат другого производителя большинство респондентов повторно назначали контроль уровня ТТГ в сыворотке крови через 8 нед (89;61,0%), а 33 (22,6%) — через 4–6 нед. В случае неизменной дозировки LT4 19 (13,0%) участников указали на отсутствие необходимости мониторинга ТТГ при переходе с одного препарата на другой, в то время как 7 (4,8%) участников предлагали оценивать клиническое состояние пациента.

В вопросах, касающихся мониторинга эффективности терапии синтетическими аналогами тиреоидных гормонов, результаты не зависели от каких-либо анамнестических (пол, возраст, стаж медицинской практики или количество пациентов, пролеченных ежегодно) характеристик участников исследования.

Лечение эутиреоидных пациентов синтетическими аналогами гормонов щитовидной железы

Ряд вопросов анкетирования учитывал отношение врачей к назначению синтетических аналогов гормонов щитовидной железы у пациентов с эутиреозом в различных ситуациях. Большая половина (84; 57,5%) респондентов утверждали, что терапия тиреоидными гормонами у таких пациентов не показана. В то же время 50 (34,2%) участников указали, что назначение LT4 может рассматриваться при женском бесплодии с высоким уровнем антител к ткани щитовидной железы, 36 (24,7%) участников отметили возможность гормональной терапии у пациентов с нетоксическим простым зобом, имеющим тенденцию к увеличению. В отдельных случаях респонденты рассматривали назначение синтетических аналогов гормонов щитовидной железы в качестве дополнительного лечения в различных клинических ситуациях, таких как устойчивая к лечению депрессия (12; 8,2%), не объяснимая усталостью (11; 7,5%), ожирение, устойчивое к изменению образа жизни (11; 7,5%) или тяжелая гиперхолестеринемия (12; 8,2%).

Комбинированная терапия LT4 и LT3

Почти половина респондентов (61; 41,8%) отметили возможность перехода на комбинированную терапию на короткий период у пациентов с некомпенсированным гипотиреозом на монотерапии LT4, а 68 (46,6%) участников никогда бы не использовали комбинированную терапию из-за низкого уровня доказательности. Только 15 (10,3%) человек рекомендовали бы комбинацию LT4+LT3 пациентам с нормальным уровнем ТТГ в сыворотке при сохранении симптомов гипотиреоза, в то время как 2 (1,4%) респондента могли бы использовать комбинированную заместительную терапию у пациентов с гипотиреозом и нормальным уровнем ТТГ в сыворотке при необъяснимом увеличении массы тела.

Результаты ответов на вопросы о комбинированной терапии не зависели от демографических параметров участников анкетирования.

Стойкие симптомы гипотиреоза у пациентов, получавших LT4

Трое (2,1%) членов БОМО «Эндокринология и метаболизм» указали, что 11–30% пациентов с гипотиреозом испытывают стойкие симптомы гипотиреоза, несмотря на наличие эутиреоза на фоне терапии LT4, в то время как 2 врача (1,4%) ответили, что более 30% таких пациентов имеют стойкие симптомы, а 17 (11,6%) респондентов не были уверены в ответе.

Согласно данным ответов большинства респондентов, доля таких пациентов составляла менее 5% (76, 52,1%) или 6–10% (48, 32,9%). Таким образом, по результатам ответов врачей, менее 10% пациентов с целевым уровнем ТТГ на заместительной терапии LT4 имеют неспецифические симптомы гипотиреоза (3,5% vs 85,5%, p<0,001).

Треть респондентов 50 (34,2%) указали на уменьшение количества таких случаев, и 51 (34,9%) участник не отмечал никаких изменений в тенденции к сохранению симптомов гипотиреоза на фоне адекватной терапии LT4; в то же время 17 (11,6%) респондентов ответили, что число пациентов увеличилось за последние 5 лет, а остальные (28; 19,2%) не уверены в ответе на данный вопрос. Результаты указанных ответов не зависели от каких-либо демографических переменных респондентов.

Учитывая сохранение неспецифических клинических симптомов гипотиреоза у ряда пациентов, несмотря на адекватную заместительную терапию, белорусским эндокринологам было предложено прокомментировать восемь возможных причин такой ситуации (табл. 3).

**Table:** Таблица 3. Оценочные результаты возможных причин наличия симптомов гипотиреоза, несмотря на адекватную заместительную терапию, согласно ответам респондентов из Беларуси, принявших участие в международном исследовании 2020 THESIS*, n (%)

Возможная причина наличия симптомов гипотиреоза	Категорически не согласен	Не согласен	Нейтрален	Согласен	Полностью согласен
Неспособность левотироксина восстановить нормальную физиологию	34 (23,3)	94 (64,4)	9 (6,1)	8 (5,5)	1 (0,7)
Психосоциальные факторы	4 (2,7)	8 (5,5)	13 (8,9)	95 (65,1)	26 (17,8)
Сопутствующие заболевания	3 (2,1)	4 (2,7)	11 (7,6)	110 (75,3)	18 (12,3)
Синдром хронической усталости	3 (2,1)	5 (3,4)	23 (15,8)	98 (67,1)	17 (11,6)
Нереалистичные ожидания пациента	8 (5,5)	15 (10,3)	35 (24,0)	69 (47,2)	19 (13,0)
Наличие аутоиммунного воспаления	14 (9,6)	54 (37,0)	51 (34,9)	23 (15,8)	4 (2,7)
В результате хронических заболеваний	3 (2,1)	4 (2,7)	18 (12,3)	110 (75,4)	11 (7,5)
В результате необходимости принимать лекарства	7 (4,8)	50 (34,2)	53 (36,3)	35 (24,0)	1 (0,7)

Таким образом, стойкие симптомы гипотиреоза на фоне заместительной терапии LT4 с достижением целевого ТТГ могут быть вызваны психосоциальными факторами, сопутствующими заболеваниями, нереалистичными ожиданиями пациента, синдромом хронической усталости, аутоиммунным воспалением, бременем хронического заболевания или необходимости приема лекарств. Лишь немногие респонденты указали, что сохранение симптомов может быть связано с неспособностью LT4 восстановить нормальную физиологию.

Добавки селена и йода

94 (64,4%) белорусских эндокринолога ответили, что добавки с селеном или йодом могут быть использованы по желанию пациента, а 18 (12,3%) указали, что такие добавки никогда не должны использоваться. 28 респондентов (19,2%) ответили, что селен или йод можно использовать у пациентов с сопутствующим аутоиммунным тиреоидитом. 26 (17,8%) участников анкетирования рекомендовали дополнительный прием йода или селена пациентам с субклиническим гипотиреозом. Данные ответов не были статистически связаны с демографическими параметрами респондентов.

Собственный опыт гипотиреоза у врачей-эндокринологов

У 18 (12,3%) участников БОМО «Эндокринология и метаболизм», заполнивших анкету, диагностирован гипотиреоз; 4 (22,0%) из числа врачей с гипотиреозом испытывали чрезмерную усталость, в то же время они не рассматривали для себя применение комбинированной терапии с использованием LT4+LT3.

Среди 120 (86,3%) респондентов, отрицавших у себя наличие гипотиреоза, 81 (64,3%) человек не рассматривал для себя комбинированную терапию либо ВЩЖ в случае диагностирования гипотиреоза. Только 8 (6,3%) участников могли бы выбрать возможность комбинированной терапии, а 43 (29,4%) врача не ответили на этот вопрос.

## ОБСУЖДЕНИЕ

Репрезентативность выборок

Участие в международном анкетировании 2020 THESIS* было первым в своем роде исследованием для белорусских врачей и позволило получить уникальные данные, отражающие реальную клиническую ситуацию.

Сопоставление с другими публикациями

Наши результаты согласуются с результатами аналогичных исследований итальянских коллег. Так, R. Negro и соавт. также оценивали приверженность врачей рекомендациям по лечению левотироксином [16]. Среди включенных в исследование 1000 врачей соответствие североамериканским (Американский колледж клинических эндокринологов, Американская тиреоидная ассоциация) и европейским (Европейская тиреоидная ассоциация) рекомендациям по ведению пациентов с гипотиреозом было зарегистрировано у 77,1 и 71,7% пациентов, а при исключении вопроса об оптимальной дозировке левотироксина — в 86,7 и 88,6% случаев [16].

В другом итальянском исследовании изучались выбор и назначение различных форм препаратов гормонов щитовидной железы эндокринологами у пациентов с эутиреозом и гипотиреозом [17]. В данном опросе приняли участие 39,3% всех итальянских эндокринологов, и 98,7% участников указали LT4 в качестве первого выбора для лечения гипотиреоза. Однако 43,2% предложили бы комбинированную терапию (LT4+LT3) пациентам с нормальным ТТГ, но стойкими симптомами. При одновременном применении нескольких препаратов, плохом биохимическом контроле гипотиреоза или плохой приверженности к лечению 81,8% эндокринологов предпочтение отдали бы жидкой форме LT4 [17].

В системном обзоре 2015 г. Е. Kraut. и соавт. были проанализированы регламентирующие руководства по заболеваниям щитовидной железы различных регионов: 6 из Северной Америки, 3 из Южной Америки и 4 из Европы [12]. Во всех документах левотироксин назван единственным рекомендованным вариантом терапии гипотиреоза. В то же время в рекомендациях Европейской тиреоидологической ассоциации экспертами прокомментирована возможность использования комбинированной терапии для краткосрочного назначения или варианта терапии у пациентов с неадекватным ответом на лечение левотироксином [12][18].

В исследовании S.J. Peterson и соавт. основную группу составили 469 пациентов, получавших LT4, клинико-лабораторные данные которых сравнили с соответствующими параметрами 9512 здоровых лиц, составивших группу контроля. Было установлено, что заместительная терапия LT4 связана с повышением на 15% уровня свободного тироксина и снижением на 15% уровня свободного трийодтиронина независимо от оптимального ТТГ [19]. Данная тенденция может быть одной из возможных причин неудовлетворенности пациентов лечением гипотиреоза LT4 [15]. С другой стороны, существуют результаты исследований, отражающие отсутствие значительного клинического эффекта при лечении субклинического гипотиреоза LT4, что может быть одной из причин недостаточной приверженности пациентов к заместительной терапии [20]. Указанные данные определяют сохранение интереса врачей к альтернативным схемам терапии сниженной функции щитовидной железы.

В настоящее время активно обсуждается проблема тканевого гипотиреоза (синдром «эутиреоидной патологии»), при котором нарушается периферическое дейодирование тироксина в трийодтиронин. Данное патологическое состояние связанно с нарушением работы 5’-дейодиназы 1-го типа, катализирующей периферическую конверсию тироксина в трийодтиронин, и 5’-дейодиназы 2-го типа. Нарушение может быть обусловлено генетически детерминированным дефектом. Существуют также результаты исследований об изменении активности дейодиназ на фоне заместительной терапии LT4. Эксперты указывают, что при нарушении метаболизма тироксина целесообразно использование комбинированной терапии [13].

Одним из направлений научных исследований является изучение использования LT4 в виде мягких гелевых капсул или жидкого препарата [10]. Новые формы, особенно в растворимом виде, могут иметь объективные преимущества в ряде клинических ситуаций, таких как заместительная терапия у детей с сопутствующей ахлоргидрией, прием нескольких лекарственных средств, парентеральное питание или состояние после бариатрической хирургии [10]. В настоящее время получены данные, что альтернативные формы LT4 могу оказывать определенное положительное влияние на качество жизни и улучшение самочувствия у пациентов со стойкими симптомами на фоне традиционного лечения таблетками [10]. Данные доводы могут быть причиной различий в поведении врачей при назначении ими различных форм выпуска препарата.

Клиническая значимость результатов

Согласно клиническим рекомендациям Российской ассоциации эндокринологов, белорусским клиническим протоколам, европейским регламентирующим документам, лечение гипотиреоза включает назначение монотерапии левотироксином натрия при значении ТТГ более 10 мЕд/л [12][21][22]. В случае субклинического гипотиреоза приоритетной для лечения группой являются беременные женщины, а возможность применения левотироксина в иных клинических ситуациях рассматривается индивидуально [18]. Отсутствие однозначной терапевтической стратегии у пациентов не с манифестным гипотиреозом обусловливает интерес данной проблеме. С учетом активно обсуждаемого в литературе, но не имеющего достаточно заслуживающих доверия научных данных в пользу существования так называемого «синдрома Уилсона» («на тироксине, но все равно плохо»), одной из задач настоящего исследования была оценка отношения врачей к данному феномену [13].

Результаты исследования показали, что левотироксин является основным препаратом, применяемым для лечения гипотиреоза в Республике Беларусь. В то же время комбинированные формы синтетических аналогов гормонов щитовидной железы и высушенный экстракт также рассматривались врачами для коррекции коморбидной патологии, а также сохраняющихся симптомов гипотиреоза, несмотря на оптимальное лечение LT4. Среди респондентов с гипотиреозом в личном анамнезе почти четверть сообщили о наличии у них слабости/утомляемости, но никто не отметил попыток применения комбинированной терапии LT3+LT4 или препаратов из ВЩЖ.

Ограничения исследования

Количество респондентов в настоящем анкетировании было относительно ограниченным и не позволяло сравнивать практику эндокринологов с практикой врачей других специальностей. Полученные результаты могли быть обусловлены недостаточным клиническим опытом применения комбинированной терапии LT3+ LT4 или препаратов из ВЩЖ, а также неоднозначными результатами зарубежных клинических исследований.

Ограничением исследования также является недоступность комбинированных препаратов тиреоидных гормонов, так же как и других форм тироксина. Поскольку такие препараты не зарегистрированы в стране, то возможность их использования врачами ограничена этим обстоятельством.

Направления дальнейших исследований

Таким образом, несмотря на широкую распространенность гипотиреоза и длительную историю применения таблетированных форм левотироксина, эффективность адекватной заместительной терапии гипотиреоза актуальна до настоящего дня. Cуществующие комбинированные препараты имеют нефизиологическое соотношение левотироксина натрия и лиотиронина. Перспективно создание комбинированных препаратов гормонов щитовидной железы пролонгированного действия в физиологическом соотношении LT4/LT3, внедрение новых лекарственных форм с соблюдением принципов персонализированной терапии.

## ЗАКЛЮЧЕНИЕ

Анализ результатов анкетирования белорусских эндокринологов, членов БОМО «Эндокринология и метаболизм», указывает на то, что методом выбора в лечении пациентов с гипотиреозом является заместительная терапия LT4. Назначение комбинированной терапии LT4+LT3 эндокринологи могут рассмотреть у пациентов с длительно не леченным гипотиреозом и у лиц со стойкими симптомами гипотиреоза, несмотря на наличие целевого значения ТГГ на фоне терапии LT4. В целом респонденты не назначают лечение LT4 пациентам с эутиреозом, однако в случае женского бесплодия с высоким уровнем тиреоидных антител высказывают возможность индивидуального назначения LT4. В различных условиях, которые могут препятствовать абсорбции LT4, белорусские эндокринологи предпочитают таблетированные формы LT4 и не ожидают существенной разницы при применении других форм лекарственного средства (мягкие гелевые капсулы, раствор).

## ДОПОЛНИТЕЛЬНАЯ ИНФОРМАЦИЯ

Источники финансирования. Работа выполнена по инициативе авторов без привлечения финансирования.

Конфликт интересов. Авторы декларируют отсутствие явных и потенциальных конфликтов интересов, связанных с содержанием настоящей статьи.

Участие авторов. Алла Шепелькевич являлась координатором международного исследования 2020 THESIS в Республике Беларусь, контролировала все этапы анкетирования, редактировала текст рукописи. Юлия Дыдышко отвечала за анализ данных, написание текста статьи. Елена Юреня являлась координатором международного исследования 2020 THESIS в республике Беларусь. Вероника Лобашова участвовала в статистической обработке данных. Аттанасио Роберто, Хегедюс Ласло, Надь Эндре, Негро Роберто, Папини Энрико, Перрос Петрос составляют научную группу международного исследования THESIS, курировавшую проведение настоящего исследования. Все авторы одобрили финальную версию статьи перед публикацией, выразили согласие нести ответственность за все аспекты работы, подразумевающую надлежащее изучение и решение вопросов, связанных с точностью или добросовестностью любой части работы.

Благодарности. Авторы статьи выражают благодарность всем членам Белорусского общественного медицинского объединения «Эндокринология и метаболизм», принявшим участие в организации и проведении настоящего исследования.
